# Cellular Zinc Deficiency Impairs Heme Biosynthesis in Developing Erythroid Progenitors

**DOI:** 10.3390/nu15020281

**Published:** 2023-01-05

**Authors:** Juyoung Kim, Jaekwon Lee, Moon-Suhn Ryu

**Affiliations:** 1 Department of Food Science and Nutrition, University of Minnesota, St. Paul, MN 55108, USA; kim00465@umn.edu; 2 Department of Biochemistry, University of Nebraska-Lincoln, Lincoln, NE 68588, USA; jlee7@unl.edu; 3 Department of Food and Nutrition, Yonsei University, Seoul 03722, Republic of Korea

**Keywords:** zinc, iron deficiency, terminal erythroid differentiation, protoporphyrin, succinylacetone, hemoglobin, anemia

## Abstract

Anemia is the most prevalent nutrition-related disorder worldwide. Zinc is an essential trace element for various biological processes in the body, and zinc deficiency has been associated with anemia in humans. However, the molecular mechanisms by which zinc availability alters red blood cell development remain uncertain. The present study identifies the essentiality of zinc during erythroid development, particularly for normal heme biosynthesis. G1E-ER4 mouse cells were used as an in vitro model of terminal erythroid differentiation, which featured elevated cellular zinc content by development. Restriction of zinc import compromised the rate of heme and α-globin production and, thus, the hemoglobinization of the erythroid progenitors. Heme is synthesized by the incorporation of iron into protoporphyrin. The lower heme production under zinc restriction was not due to changes in iron but was attributable to less porphyrin synthesis. The requirement of adequate zinc for erythroid heme metabolism was confirmed in another erythropoietic cell model, MEL-DS19. Additionally, we found that a conventional marker of iron deficiency anemia, the ZnPP-to-heme ratio, responded to zinc restriction differently from iron deficiency. Collectively, our findings define zinc as an essential nutrient integral to erythroid heme biosynthesis and, thus, a potential therapeutic target for treating anemia and other erythrocyte-related disorders.

## 1. Introduction

According to the World Health Organization (WHO), anemia is defined as low hemoglobin levels or red blood cell count and can be caused by nutritional deficiencies in iron, folate, and vitamin B12; by infection and chronic inflammation; or by genetic hemoglobin disorders [1]. Although nutrition-based strategies have been demonstrated effective in the prevention of nutritional anemia [1], the global prevalence of anemia remains high, affecting approximately 23% of the population [2]. Anemia is the most common disorder attributable to poor nutrition worldwide and is especially prevalent in women of reproductive age and children under 5 years [2].

In humans, erythrocytes in circulation are constantly turned over, with an approximate lifespan of 120 days. Erythrocyte homeostasis of the body is maintained by the supply of new red blood cells, mainly from steady-state erythropoiesis in the bone marrow. The spleen and liver can become erythropoietic sites for extramedullary erythropoiesis under certain pathological conditions requiring a rapid expansion in red cell production, such as acute hemolytic anemia [3]. Erythroid progenitors mature into erythrocytes via committed erythropoiesis (i.e., terminal erythroid differentiation) upon stimulation by erythropoietin (EPO). EPO activates the production of proteins integral to iron assimilation, heme biosynthesis, and remodeling of cellular structure [4]. Disruption in these molecular events leads to failure of normal terminal erythroid differentiation and, thus, anemia at the organismal level. Of relevance is iron deficiency anemia attributed to impaired heme biosynthesis.

Erythroid progenitors undergo dynamic nutrient demand and composition changes during terminal erythroid differentiation. For instance, iron is imported early, transiently stored, and then utilized for heme biosynthesis during development [5]. Zinc is the second most abundant trace element in the erythron after iron [6,7]. Recently, intracellular zinc of erythroid progenitors was identified as a molecular switch driving erythroid development and cell survival and thus is under tight regulation [8]. Additionally, zinc serves as a catalytic or structural component for metalloproteins critical to red cell metabolism, including δ-aminolevulinic acid dehydratase (ALAD) and superoxide dismutase 1 [9,10,11]. These proteins, respectively, are key to heme biosynthesis and defense against oxidative stress in cells of the erythron. These findings identify the biological significance of zinc in maintaining the amount and integrity of red blood cells.

Zinc is a type 2 essential trace element nutrient involved in various molecular and biological processes of the human body [12]. Its deficiency remains highly prevalent, partly due to the lack of a specific deficiency symptom and status assessment tool for zinc, with an estimated global prevalence as high as 16% worldwide [13,14]. Several preclinical studies have identified the causal effect of dietary zinc restriction on molecular indices of impaired erythropoiesis [15,16]. Additionally, the epidemiological association of serum zinc concentrations with the risk of anemia has been reported in humans [17,18,19], and symptoms of iron deficiency anemia have been included in clinical outcomes of poor zinc nutrition cases [20,21]. Although the potential role of zinc status in anemia risk has been recognized, further mechanistic work is needed to establish the nutritional implications of zinc status in red cell metabolism.

The primary aim of the present study was to elucidate the role and essentiality of zinc in developing erythroid progenitors particularly for their heme biosynthesis, a process integral to maturation into red blood cells. Mouse erythroid cell line G1E-ER4 cells were used as an extensively validated cell culture model of EPO-induced terminal erythroid differentiation and were complemented by an alternative mouse model of induced heme biosynthesis, MEL-DS19 cells. Hemoglobinization of both cell models was significantly impaired when cellular zinc availability became restricted and produced responses in porphyrin metabolites, including zinc protoporphyrin (ZnPP) and free protoporphyrin IX (PPIX), different from those produced by iron restriction.

## 2. Materials and Methods

### 2.1. Cell Culture and Differentiation

The G1E-ER4 cell line and MEL-DS19 cells were obtained from Dr. Mitchell Weiss (St. Jude Children’s Research Hospital, Memphis, TN, USA) and Dr. Caroline Philpott (National Institutes of Health, Bethesda, MD, USA), respectively. The G1E-ER4 cells were cultured in Iscove’s modified Dulbecco’s medium (IMDM) supplemented with 15% fetal bovine serum, 100 U/mL penicillin-streptomycin, monothioglycerol (1:10,000), 2 U/mL EPO (Epogen; Amgen, Thousand Oaks, CA, USA), and conditioned medium from Kit ligand-producing Chinese hamster ovary (CHO) cells (1:200), as previously described [5]. To induce erythroid differentiation, cells at a density of 0.2 × 10^6^ cells/mL were treated with 100 nM β-estradiol (β-Est; Sigma-Aldrich, St. Louis, MO, USA) and cultured for up to 48 h. MEL-DS19 cells were grown in Dulbecco’s modified eagle medium (DMEM) containing 10% heat-inactivated FBS, 2 mM glutamine, and 1% MEM non-essential amino acids solution (MEM NEAA) [22]. The cells were maintained at 0.05–0.8 × 10^6^ cells/mL and incubated at 37 °C in 5% CO_2_. To induce hemoglobinization of the MEL cells, the cells were seeded at a density of 5 × 10^4^ cells/mL and cultured with 1.5% dimethyl sulfoxide (DMSO; Sigma-Aldrich) for up to 5 days.

### 2.2. Chemical Treatments and Cell Viability

Zinc availability was restricted by adding diethylenetriamine pentaacetate (DTPA; Sigma-Aldrich) or *N*,*N*,*N*′,*N*′-tetrakis(2-pyridylmethyl)-ethylenediamine (TPEN; Sigma-Aldrich) at the final concentrations of 50 and 10 μM, respectively. Zinc replenishment against DTPA treatment was achieved by adding equimolar zinc chloride. For G1E-ER4 experiments, cellular iron availability was limited by deferoxamine (DFO; Sigma-Aldrich) at a final concentration of 50 μM, and succinylacetone (SA; Sigma-Aldrich), an ALAD inhibitor [23], was treated at a concentration of 0.2 mM twice with a 24 h interval. For MEL-DS19 experiments, 50 μM DTPA, 25 μM DFO, or 1 mM SA was added to the medium 2 days after the DMSO treatment. Cell density and viability were determined using an automated cell counter R1 (Olympus, Shinjuku City, Tokyo, Japan) after mixing the cells with an equal volume of 0.4% trypan blue.

### 2.3. Quantitative Metal Analyses

The cells were washed twice with ice-cold PBS containing 10 mM ethylenediaminetetraacetic acid (EDTA) and transferred to acid-washed tubes at the third washing step. Frozen pellets of a known number of cells were processed and analyzed at the University of Nebraska for quantitative measurements of mineral contents using inductively coupled plasma mass spectrometry (ICP-MS), as previously described [24]. Metal contents were normalized to total protein contents determined by the bicinchoninic acid (BCA) assays (Thermo Scientific, Waltham, MA, USA; #23225), cell counts, or phosphorous levels of each sample.

### 2.4. Heme and Total Porphyrin Assays

For cellular heme assays, the cells were treated with an NP-40-based lysis buffer containing 100 mM Tris-HCl (pH 7.5), 50 mM KCl, 0.1% NP-40, 5.0% glycerol, and protease inhibitor cocktail (Roche, Basel, Switzerland; #11836170001). The heme content in the cell lysates was colorimetrically determined by the QuantiChrom heme assay (BioAssay Systems, Hayward, CA, USA; DIHM-250), following the manufacturer’s protocol, and normalized to the number of cells. For total porphyrin quantitation [25], the cell lysates were treated with nine volumes of a mixture of ethyl acetate and acetic acid (at 4:1, *v*/*v*) and centrifuged at 1000× *g* for 3 min to precipitate proteins. The supernatant was collected, treated with an equal volume of 1.5N HCl, and centrifuged at 1000× *g* for 3 min. The clear top layer was isolated and analyzed by spectrofluorometry with excitation at 405 nm and measures of emission at 604 nm. ZnPP and free PPIX concentrations were determined as previously described [26,27]. Briefly, the cell lysates were mixed with nine volumes of ethanol and centrifuged at 20,000× *g* for 10 min. Emissions at 589 and 623 nm by excitation at 415 nm were measured using a spectrofluorometer for ZnPP and free PPIX quantitation, respectively. Serial dilutions of the cell lysates or ZnPP (Thermo Scientific; 032068) were prepared and analyzed as standards, and the final measures were normalized to the cell counts.

### 2.5. Quantitative Real-Time PCR (qPCR)

The cells were treated with a TRI reagent (Sigma-Aldrich; #T9424) and processed for total RNA isolation using the Direct-zol RNA Mini Preparation Kit (Zymo Research, Irvine, CA, USA; R2050) by adding the TRI reagent according to the manufacturer’s instructions or by adding 1-bromo-3-chloropropane. The cDNA was reverse-transcribed using the High-Capacity cDNA Reverse Transcription Kit (Applied Biosystems, Waltham, MA, USA; #4368814). The transcripts were PCR-amplified using Power SYBR Green Master Mix (Applied Biosystems; #A25742) and detected using a CFX Connect Real-Time System (Bio-Rad). The relative mRNA abundance was determined by the 2^−ΔΔ*C*t^ method. Primers specific to each gene of interest were designed using Primer-BLAST [28] and are provided in Table 1.

### 2.6. Western Blot Analyses

The cells were lysed in the Pierce RIPA buffer (Thermo Scientific; #89900) supplemented with a protease inhibitor cocktail (Roche; #11836170001), and the protein contents of the lysates cleared by centrifugation at 12,000× *g* for 5 min were determined using the Pierce BCA Protein Assay Kit (Thermo Scientific; #23225). For immunoblotting, equal amounts of protein were prepared with a Laemmli buffer (Bio-Rad; #1610747) containing 2.5% β-mercaptoethanol and separated by 4–20% polyacrylamide gel electrophoresis (PAGE) in SDS containing a Tris−glycine running buffer. The separated protein was transferred to the nitrocellulose membrane using the Trans-Blot Turbo Transfer System (Bio-Rad). Immunoblotting was conducted using the following antibodies: mouse anti-metallothionein 1 (MT1; 1:1000; Invitrogen, Waltham, MA, USA; MA 1-25479), rabbit anti-hemoglobin-alpha (HBA; 1:1000; Proteintech, Rosemont, IL, USA; 14537-1-AP), rabbit anti-iron regulatory protein 2 (IRP2; 1:1000; Dr. Betty Leibold, University of Utah), and anti-glyceraldehyde 3-phosphate dehydrogenase (GAPDH; 1:2000; Bio-Rad; 12004167) primary antibodies. The subsequent probing was processed with NIR fluorescent secondary antibodies (1:10,000; Li-Cor, Lincoln, NE, USA) and visualized using a Li-Cor Odyssey Fc detection system.

### 2.7. Statistical Analyses

All the data are presented as mean ± standard deviation. Depending on the experimental design, Student’s *t*-test or one-way ANOVA, two-way ANOVA, or two-way repeated-measure ANOVA with Dunnett’s or Tukey’s HSD post hoc test was conducted to determine statistically significant differences. A *p*-value < 0.05 was considered statistically significant.

## 3. Results

### 3.1. G1E-ER4 Cells Acquire Zinc during Development and Depend on Zinc for Survival

**Figure 1:**
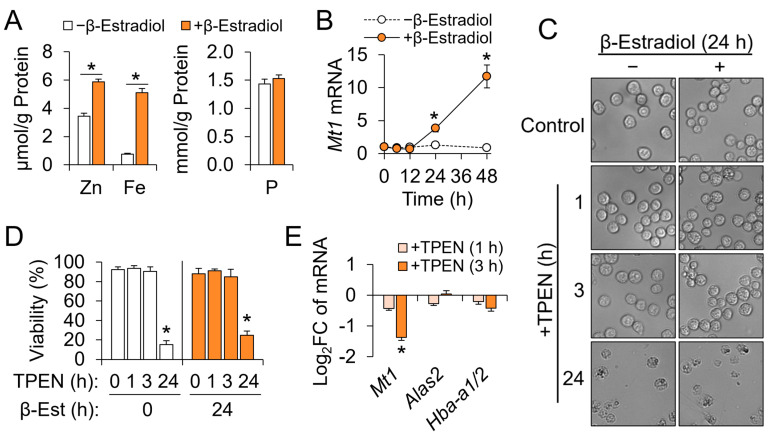
G1E-ER4 erythroid progenitors accumulate zinc during differentiation and require zinc for survival. The differentiation was induced by adding β-estradiol (β-Est). (**A**) Accumulations of total cellular zinc and iron in developing G1E-ER4 cells 48 h after terminal erythroid differentiation. Cellular contents were normalized to total protein contents (*n* = 4). (**B**) Increased *Mt1* transcript abundance by erythroid development, normalized to *Tbp* (*n* = 3). (**C**) Representative cell microscopic images of G1E-ER4 cells treated with or without 10 μM TPEN. β-Est treatments were for 24 h. (**D**) Effects of TPEN on viability of G1E-ER4 cells treated with or without β-Est (*n* = 4). (**E**) Effects of TPEN on *Mt1*, *Alas2*, and *Hba-a1/2* transcript abundance normalized to *Tbp* (*n* = 3). Data presented as mean ± SD. * *p* < 0.05. β-Est, β-estradiol; TPEN, *N,N,N′,N′*-tetrakis(2-pyridylmethyl)-ethylenediamine.

G1E-ER4 cells undergo committed erythropoiesis by β-Est treatment and have been extensively characterized as an in vitro model of terminal erythroid differentiation [5]. To determine whether zinc has a role in terminal erythroid differentiation, we determined the mineral contents using ICP-MS of cells with or without β-Est treatment. After 48 h of differentiation, the cellular zinc contents increased 1.7-fold and were at a molar concentration comparable to that of iron (Figure 1A). In contrast to zinc and iron, cellular phosphorus contents were not affected by the differentiation of the erythroid progenitors. Metallothionein (Mt1) functions as a cytosolic buffer for zinc fluctuation and responds to zinc at the transcript level [29,30]. In agreement with the zinc measures, G1E-ER4 cells featured a gradual increase in *Mt1* transcript abundance by differentiation (Figure 1B). These results suggest that erythroid progenitors assimilate zinc during maturation to support its role in red cell development.

Next, we restricted the zinc availability in the G1E-ER4 erythroid progenitors using a cell-permeable zinc chelator, TPEN, to determine the effects of zinc depletion in the erythroid progenitors. Both cells treated without (proliferating) and with β-Est for 24 h (developing) featured apparent changes in cell morphology and density when incubated with TPEN for 24 h (Figure 1C). Cell viability by trypan blue exclusion decreased significantly after 24 h treatment with TPEN. While TPEN treatments for 1 and 3 h were neither cytotoxic nor morphologically altered the cells (Figure 1D), they reduced the *Mt1* mRNA abundance, confirming the effectiveness of TPEN in depleting the cellular zinc pool. Expression of erythropoietic genes, 5’-aminolevulinate synthase (*Alas2*), and hemoglobin alpha, adult chain 1/2 (*Hba-a1/2*) in differentiating G1E-ER4 cells was not affected by TPEN-induced zinc depletion (Figure 1E).

### 3.2. Restriction of Cellular Zinc Impairs Hemoglobinization G1E-ER4 Cells by Differentiation

**Figure 2:**
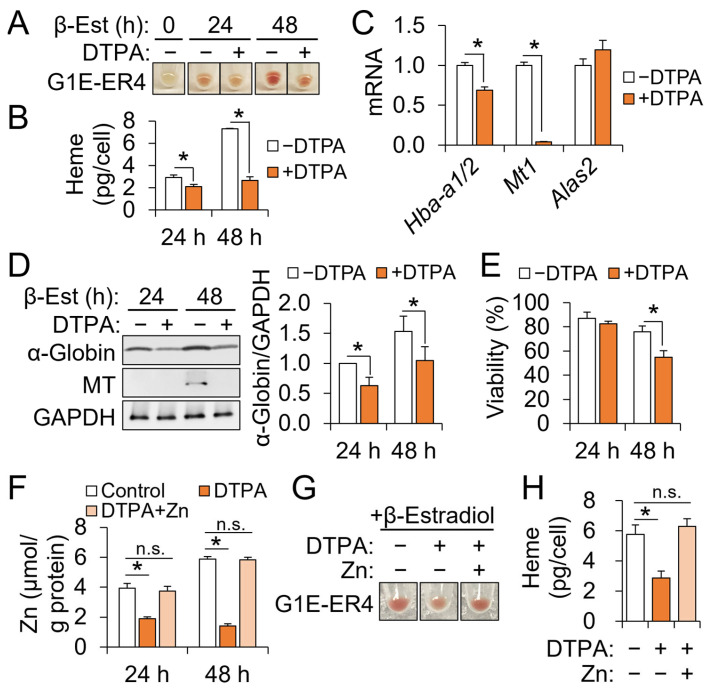
Mild cellular zinc restriction impairs heme biosynthesis by erythroid progenitor without compromised cell viability. (**A**) Representative cell pellet images of G1E-ER4 cells differentiating under zinc restriction by DTPA. (**B**) Decreased heme contents by DTPA in developing G1E-ER4 cells (*n* = 3). (**C**) Effects of DTPA on *Mt1*, *Hba-a1/2*, and *Alas2* transcripts at 24 h of differentiation, normalized to *Tbp* (*n* = 3). (**D**) Responses of α-globin and metallothionein protein abundance to zinc restriction in differentiating G1E-ER4 cells (*n* = 4). (**E**) Viability of G1E-ER4 cells with or without DTPA treatment for 24 and 48 h (*n* = 3). (**F**–**H**) Restoration of cellular zinc levels (**F**), hemoglobinization (**G**), and heme contents (**H**) of DTPA-treated G1E-ER4 cells by equimolar zinc (50 μM) treatment (*n* = 4). Cells were treated with β-estradiol (β-Est) for differentiation and DTPA or zinc for each zinc condition at 0 h, and cultured for 24 or 48 h. Cell pellet images and heme data were collected 48 h after zinc treatments and differentiation (*n* = 4). Data presented as mean ± SD. * *p* < 0.05. β-Est, β-estradiol; DTPA, diethylenetriaminepentaacetic acid; n.s., not significant.

The effects of zinc restriction on erythroid development could not be assessed using the TPEN model, primarily because the cells could not survive long enough to carry out heme biosynthesis. Thus, we used a cell-impermeable zinc chelator, DTPA, to restrict the extracellular zinc availability and, thus, its import. Zinc restriction by DTPA led to less intense red coloration, indicative of impaired hemoglobinization, by differentiation of the G1E-ER4 cells (Figure 2A). A quantitative assessment of cellular heme contents confirmed lower heme production by DTPA-induced zinc restriction (Figure 2B). Decreases in *Mt1* and *Hba-a1/2* transcript abundance reflected the decrease in cellular zinc and heme contents by DTPA, respectively (Figure 2C), and were confirmed at the protein level by western blot analyses (Figure 2D). Notably, DTPA did not affect the expression of *Alas2*, the rate-limiting enzyme for erythroid heme biosynthesis (Figure 2C), indicating that zinc restriction did not universally affect the expression of erythropoietic genes by terminal erythroid differentiation. Unlike TPEN, DTPA did not compromise cell viability after 24 h of treatment (Figure 2E) when impaired hemoglobinization by zinc restriction was initially observed (Figure 2A).

DTPA can potentially chelate other trace elements and thus contribute to the observed heme deficiency via a zinc-independent mechanism. Iron, which is integral to erythroid development and heme biosynthesis, was of particular concern in our study. To confirm that the observed effects of DTPA were due to the loss of bioavailable zinc, we assessed whether cells could be rescued from the DTPA effects by adding back zinc. Using ICP-MS, we confirmed that equimolar zinc recovered the zinc contents of DTPA-treated cells up to the control level (Figure 2F). Additionally, impaired hemoglobinization (Figure 2G) and heme deficiency (Figure 2H) by DTPA were completely corrected by the addition of zinc to DTPA-treated cells. Thus, the DTPA effects on heme production by erythroid development were due to the restriction of zinc availability rather than any other trace element potentially affected by the DTPA treatment.

### 3.3. Cellular Iron Contents of Differentiating G1E-ER4 Cells Are Not Affected by Zinc Status

**Figure 3:**
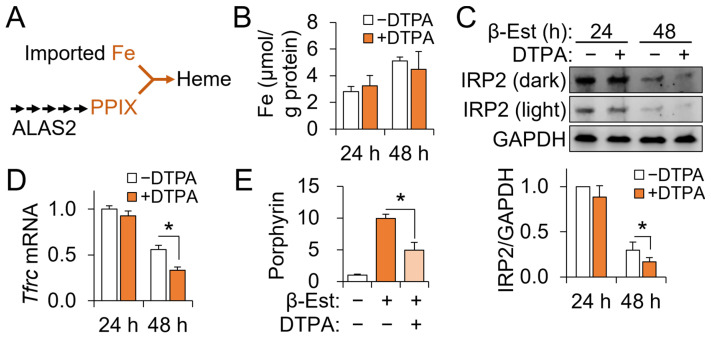
Cellular zinc restriction impairs porphyrin production by developing G1E-ER4 cells without changing cellular iron contents. (**A**) Simple scheme of the heme biosynthetic pathway. (**B**) Lack of changes in cellular iron contents of developing G1E-ER4 cells by DTPA. Cellular contents were normalized to total protein (*n* = 4). (**C**) IRP2 protein abundance in response to differentiation induction and DTPA treatments in G1E-ER4 cells (*n* = 3). Dark and light indicate images captured at higher and lower intensity, respectively. (**D**) Changes in *Trfc* mRNA abundance, a regulatory target of IRP2, by differentiation and DTPA (*n* = 3). (**E**) Reduction in total porphyrin contents by DTPA in differentiating G1E-ER4 cells. Total porphyrin contents were determined after 48 h of treatments and normalized to cell numbers. Data presented as mean ± SD. * *p* < 0.05. β-Est, β-estradiol; DTPA, diethylenetriaminepentaacetic acid; PPIX, protoporphyrin IX.

Heme biosynthesis is completed via the incorporation of iron into PPIX by ferrochelatase activity (Figure 3A). Thus, we questioned if impaired heme production by zinc restriction is attributable to either low iron availability or impaired PPIX biosynthesis. First, we determined the total cellular iron contents using ICP-MS, which showed that the contents were not affected by DTPA treatment (Figure 3B), thus indicating that zinc status did not influence cellular capacity to retain iron. Impaired conversion of nonheme iron to heme iron is expected to result in the accumulation of nonheme iron within cells, particularly when the total iron content does not change. Nonheme iron within the cytosol contributes to the labile iron pool [31], and expansion of the labile iron pool facilitates the turnover of IRP2 [32]. IRP2 protein abundance declined between 24 and 48 h of differentiation in both zinc-adequate and -deficient G1E-ER4 cells, supporting an expansion of the cytosolic nonheme iron pool by development per se (Figure 3C). IRP2 was not affected by zinc status at 24 h of differentiation but became significantly lower in zinc-restricted cells at the 48-h time-point compared to their corresponding control cells. Transferrin receptor protein 1 (*Tfrc*) transcripts are stabilized by the binding of IRP2 and thus were quantified as an indicator of IRP2 activity. The effects of differentiation and zinc status on *Tfrc* mRNA abundance resembled the expression pattern of the IRP2 protein (Figure 3D). Collectively, the decreases in IRP2 protein and *Tfrc* transcript abundance after 48 h of zinc restriction suggest an expansion of the cellular labile iron pool, potentially due to less efficient conversion of nonheme iron into heme.

Having established that the cellular iron contents did not explain the lower heme contents of the zinc-restricted G1E-ER4 cells, we next determined the effect of DTPA on cellular protoporphyrin production by measuring the total porphyrin. Erythroid porphyrins include heme as the final product, ZnPP as the byproduct, and free PPIX as an intermediate, all of which are produced via the heme biosynthetic pathway. Unlike iron, the total porphyrin level was significantly lower in the DTPA-treated cells (Figure 3E), implying that impaired PPIX production caused heme deficiency of cells differentiating under zinc restriction.

### 3.4. Zinc Restriction Differentially Affects Porphyrin Metabolites Produced during Hemoglobinization of MEL-DS19 Cells

**Figure 4:**
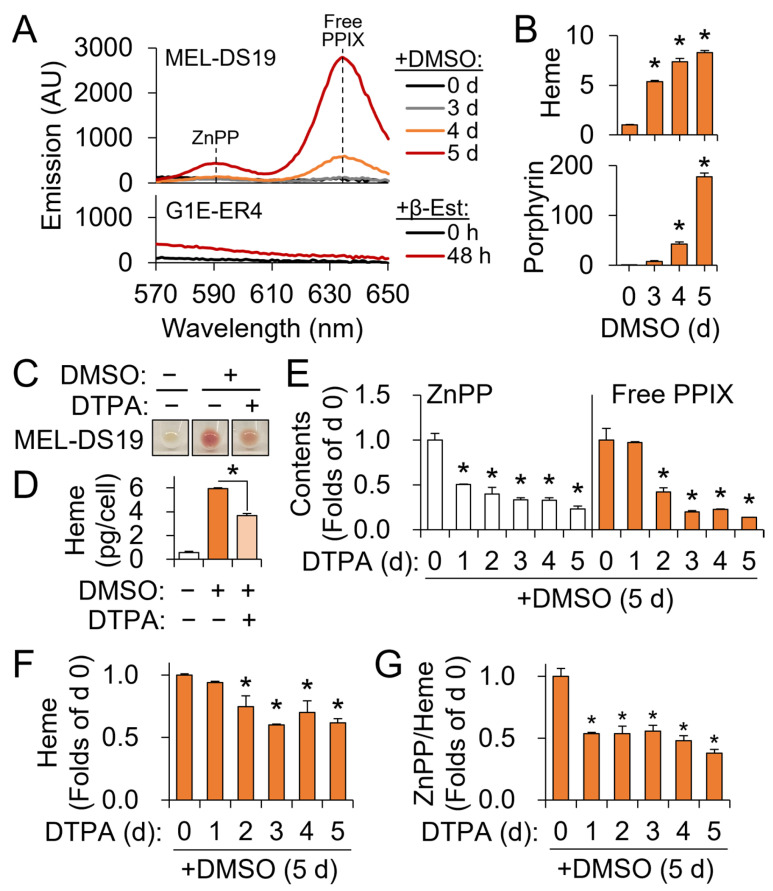
Zinc restriction results in lower heme, zinc protoporphyrin, and free protoporphyrin production by MEL-DS19 cells induced for hemoglobinization. (**A**) Fluorescence spectra of free PPIX and ZnPP in MEL-DS19 cells treated with DMSO and G1E-ER4 cells treated with β-Est. (**B**) Accumulation of heme and porphyrin in DMSO-treated MEL-DS19 cells (*n* = 3), shown in folds of day 0. (**C**) Cell pellet images of MEL-DS19 cells with impaired hemoglobinization by DTPA-induced zinc restriction. (**D**) Zinc limitation by DTPA compromises DMSO-induced heme production in MEL-DS19 cells. Cells were treated with or without DMSO and DTPA for 5 days. (**E**) Effects of zinc restriction on ZnPP and free PPIX production by MEL-DS19 cells treated with DMSO for 5 days. Contents were normalized to the number of cells (*n* = 3). (**F**,**G**) Heme contents (**F**) and changes in ZnPP/heme (**G**) by the conditions of (**E**). Data presented as mean ± SD. * *p* < 0.05. DMSO, dimethyl sulfoxide; ZnPP, zinc protoporphyrin; PPIX, protoporphyrin IX.

To improve understanding of how cellular zinc status affects porphyrin metabolism, the abundance of ZnPP and free PPIX contents was assessed. For this purpose, we used MEL-DS19 cells, which undergo hemoglobinization upon induction by DMSO [22]. This choice was primarily due to the lack of detectable levels of ZnPP and free PPIX in the G1E-ER4 cells (Figure 4A). Differentiating G1E-ER4 cells represent the erythroid lineages from proerythroblasts to orthochromatic erythroblasts and thus only recapitulate the relatively early stages of terminal erythroid development [5,33]. This may explain the difficulty of detecting quantitatively meaningful levels of cellular ZnPP and free PPIX. When using MEL-DS19 cells, spectral peaks corresponding to ZnPP and free PPIX became prominent after 4 and 5 days of DMSO treatment, allowing quantitative assessments of these porphyrin species (Figure 4A). Notably, the rate of total porphyrin synthesis peaked between days 4 and 5 of the DMSO treatment, while heme contents only gradually increased during this time (Figure 4B). This indicates that MEL-DS19 cells start accumulating nonheme porphyrin molecules, such as free PPIX and ZnPP, after 4 days of hemoglobinization, as shown in Figure 4A.

Zinc restriction by DTPA impaired hemoglobinization (Figure 4C) and heme biosynthesis in DMSO-treated MEL-DS19 cells (Figure 4D). These findings confirm the essentiality of zinc for normal red cell development and heme biosynthesis initially characterized using G1E-ER4 cells. Next, we determined how zinc restriction affects the production of ZnPP and free PPIX. ZnPP in MEL-DS19 was significantly reduced by DTPA as early as 1 day after treatment (Figure 4E). By contrast, free PPIX contents were lowered by zinc restriction after a delayed response, specifically when treated with DTPA for at least 2 days (Figure 4E). Similarly, it took more than 2 days of DTPA treatment for zinc restriction to repress heme production (Figure 4F). These observations indicate that the formation of ZnPP, attributable to the misincorporation of zinc into PPIX [34], is quickly prevented by zinc restriction, while impairment of PPIX production by zinc restriction requires zinc depletion that is long enough, for instance, to influence the activity of a protein mediating its biosynthetic pathway.

### 3.5. Erythroid Progenitors Developing under Zinc Restriction Manifest Changes in Heme Metabolites Distinct from Those of Iron Deficiency

The erythroid ZnPP/heme ratio is a sensitive diagnostic biomarker for iron deficiency anemia [35,36]. Our in vitro experiments revealed a decrease in the ZnPP/heme ratio by zinc restriction (Figure 4G), a mode of response opposite to that expected by iron deficiency. The responsiveness of the ZnPP/heme ratio to zinc restriction was acute and steady throughout the studied time course (Figure 4G).

**Figure 5:**
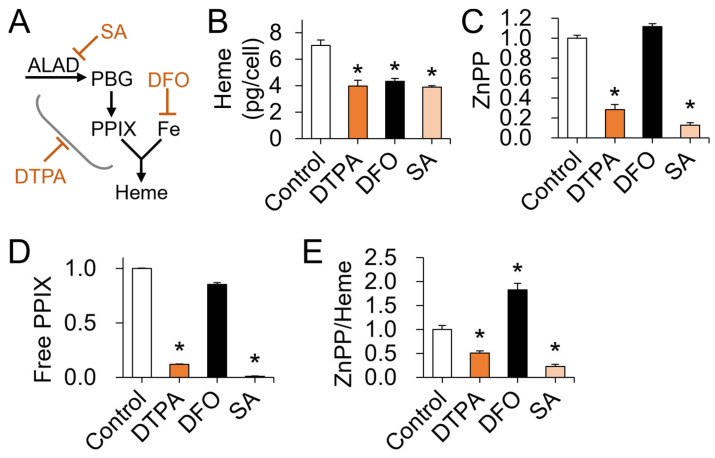
Erythroid zinc restriction and iron deficiency display distinct heme metabolite profiles. (**A**) A scheme of the effects of DTPA, succinylacetone (SA), and deferoxamine (DFO) on heme biosynthesis. (**B**) Impaired heme production by DTPA (50 μM), DFO (25 μM), and SA (1 mM) for 2 days in MEL-DS19 cells. Cells were treated with DMSO for 5 days to induce hemogloninization. (*n* = 3). (**C**–**E**) Changes in ZnPP (**C**), free PPIX (**D**), and ZnPP/heme ratio (**E**) by DTPA, DFO, or SA in DMSO-treated MEL-DS19 cells (*n* = 3). Data presented as mean ± SD. * *p* < 0.05. DMSO, dimethyl sulfoxide; ZnPP, zinc protoporphyrin; PPIX, protoporphyrin IX.

Our earlier data indicate that zinc restriction leads to lower PPIX production without changes in cellular iron contents (Figure 3). Thus, we next determined how iron restriction by DFO or limiting PPIX synthesis by an ALAD inhibitor SA differently or similarly affects the ZnPP/heme ratio using MEL-DS19 cells (Figure 5A). DTPA, DFO, and SA treatments of the MEL-DS19 cells produced a comparable decline in the cellular heme contents (Figure 5B). Notably, ZnPP and free PPIX contents were significantly lower when zinc was restricted (Figure 5C,D). Inhibition of ALAD by SA affected ZnPP and PPIX likewise. However, cellular iron restriction by DFO only affected cellular heme contents, which contributed to an increase in the ZnPP/heme ratio compared to the control level (Figure 5E). In contrast to iron restriction, deficiency in zinc or ALAD activity resulted in a lower ZnPP/heme ratio compared to the control. Overall, our findings indicate that zinc restriction influences porphyrin metabolism in a manner similar to the inhibition of ALAD and that a decrease in the ZnPP/heme ratio could potentially function as a biomarker differentiating impaired heme biosynthesis by zinc deficiency from that by limited iron availability.

## 4. Discussion

Approximately 1.1 billion people are at high risk of zinc deficiency worldwide [13]. Anemia, defined as a low red blood cell count or hemoglobin content, is another major public health concern [2]. Plasma and serum zinc have been proposed as a predictor for hemoglobin concentrations and anemia risk in clinical studies, independent of iron status [17,18,19,37]. These findings emphasize the importance of zinc in understanding the etiology of anemia. Our experiments directly address the requirement of zinc in red cell development and reveal the implications of zinc deficiency for heme metabolism, particularly protoporphyrin synthesis, during terminal erythroid differentiation.

Circulating mature erythrocytes contain approximately 10 times higher zinc concentration compared to plasma or serum [38], implying a physiological need for this nutrient on the part of erythroid cells. A few animal studies have demonstrated the demand for zinc in red blood cell production and functioning. In the bone marrow of rats, zinc assimilation was observed in the condition of increased erythropoietic demand due to hemolytic anemia [3]. Redistribution of zinc was reported in anemic mice and rats, leading to elevated bone marrow and erythrocyte zinc [39]. Additionally, upregulation of the zinc importer ZIP10 and downregulation of the exporter ZnT1 in the plasma membrane fraction of mature red blood cells by dietary zinc restriction have been demonstrated in mice [40]. These observations support our finding that erythroid cells have a physiological demand for zinc, particularly for adequate heme production during development into mature red cells.

The incorporation of iron completes heme biosynthesis into PPIX by ferrochelatase activity. Thus, a reduction in either iron or PPIX availability is expected to compromise heme production. Impaired hemoglobinization by restriction of the cellular zinc supply was attributable to inadequate production of porphyrin by developing erythroid progenitors. Notably, zinc has been characterized as a catalytic cofactor for ALAD, the first cytosolic enzyme in the heme biosynthetic pathway. ALAD catalyzes the conversion of δ-aminolevulinic acid to porphobilinogen [9,10]. Porphobilinogen is eventually oxidized to PPIX, which is converted into heme. In rats, the consumption of a zinc-deficient diet lowered erythroid ALAD activity [41]. Moreover, ALAD activity in red blood cells showed a response to the intake of zinc supplementation in humans [42]. Thus, the lower levels of porphyrin produced by zinc-deficient erythroid cells may be attributable to the zinc dependence of their ALAD activity.

Anemia is clinically defined as a condition resulting in fewer red blood cells in circulation and lower hemoglobin content in the blood [2]. Thus, either lower numbers of total red cells or a decline in mean cell hemoglobin contents contributes to the development of anemia. Cellular zinc import, particularly by ZIP8, has been identified as essential for the survival of erythroid progenitors during their maturation [8], as demonstrated by the TPEN-treated G1E-ER4 cells. Loss in developing erythroid progenitors by impaired cellular zinc acquisition may lead to lower erythrocyte production via erythropoiesis. Notably, our experiments identified impaired hemoglobinization before any losses in cell count, viability, or cell size caused by zinc restriction. These findings indicate that poor zinc nutrition may contribute to the development of anemia by either reducing the number of newly produced red blood cells via erythropoiesis or by impairing heme production by each erythroid progenitor. Whether poor zinc nutrition influences erythroid cell number or cellular heme content to different degrees in live animals or humans warrants further investigation via relevant in vivo models of dietary zinc deficiency.

A reliable biomarker or status indicator for zinc deficiency is lacking [43]. In a clinical setting, hemoglobin content and erythrocyte morphology or size are commonly assessed as indices for nutrient status—for example, iron or vitamin B12 deficiency, which causes microcytic and macrocytic anemia, respectively [44]. Additionally, iron deficiency anemia can be defined by an increase in the ZnPP/heme ratio [34], which is caused by the relative increase in zinc as a substrate for ferrochelatase when cells become iron-restricted [34]. In the present study, we demonstrate that ZnPP contents decrease due to zinc restriction in cells, resulting in a decline in the ZnPP/heme ratio. Thus, a low ZnPP/heme ratio could serve as a potential diagnostic approach to differentiate iron deficiency anemia from anemia caused by zinc deficiency.

## 5. Conclusions

Our findings mechanistically demonstrate the requirement of adequate zinc supply to developing erythroid cells to support their proliferation and survival [8] and facilitate their core metabolic process, heme production. To our knowledge, this is the first study to directly address the essentiality of zinc for normal erythroid heme metabolism and hemoglobinization. Based on these findings, future in vivo animal or clinical studies should aim to determine the essentiality of dietary zinc intake for maintaining systemic erythrocyte homeostasis and preventing anemia. Anemia remains a primary nutrition disorder worldwide [2], and many cases remain ineffectively treated due to their unclear etiology. Our findings identify improving the nutritional status of zinc as a potential therapeutic approach for correcting these anemia cases.

## Figures and Tables

**Table 1:** qPCR Primer Sequences.

Gene	Primer Set	Sequence
*Mt1*	Forward	5′-CCTCCTGCAAGAAGAGCTGC-3′
	Reverse	5′-TTCGTCACATCAGGCACAGC-3′
*Alas2*	Forward	5′-CAGAGGGCAGCTCCAGAAGTT-3′
	Reverse	5′-GCTTCGGGTGGTTGAATCC-3′
*Hba-a1/2*	Forward	5′-CGTGCTGACCTCCAAGTACC-3′
	Reverse	5′-GGTACAGGTGCAAGGGAGAG-3′
*Tfrc*	Forward	5′-TCACTTCCTGTCGCCCTATGT-3′
	Reverse	5′-AGAGTGTGAGAGCCAGAGCC-3′
*Tbp*	Forward	5′-AGTTGTGCAGAAGTTGGGCT-3′
	Reverse	5′-TACTGAACTGCTGGTGGGTCA-3′

## Data Availability

The data presented in this study are available on reasonable request from the corresponding author.

## References

[1] World Health Organization (2017). Nutritional Anaemias: Tools for Effective Prevention and Control.

[2] Gardner W., Kassebaum N. (2020). Global, Regional, and National Prevalence of Anemia and Its Causes in 204 Countries and Territories, 1990–2019. Curr. Dev. Nutr..

[3] Huber K.L., Cousins R.J. (1993). Zinc Metabolism and Metallothionein Expression in Bone Marrow during Erythropoiesis. Am. J. Physiol.-Endocrinol. Metab..

[4] Koury S., Yarlagadda S., Moskalik-Liermo K., Popli N., Kim N., Apolito C., Peterson A., Zhang X., Zu P., Tamburlin J. (2007). Differential Gene Expression during Terminal Erythroid Differentiation. Genomics.

[5] Ryu M.-S., Zhang D., Protchenko O., Shakoury-Elizeh M., Philpott C.C. (2017). PCBP1 and NCOA4 Regulate Erythroid Iron Storage and Heme Biosynthesis. J. Clin. Investig..

[6] Monacelli R., Tanaka H., Yoe J.H. (1956). Spectrochemical Determination of Magnesium, Chromium, Nickel, Copper and Zinc in Human Plasma. Clin. Chim. Acta.

[7] Walther L.E., Winnefeld K., Sölch O. (2000). Determination of Iron, Copper, Zinc, Magnesium and Selenium in Plasma and Erythrocytes in Neurosurgical Patients. J. Trace Elem. Med. Biol..

[8] Tanimura N., Liao R., Wilson G.M., Dent M.R., Cao M., Burstyn J.N., Hematti P., Liu X., Zhang Y., Zheng Y. (2018). GATA/Heme Multi-Omics Reveals a Trace Metal-Dependent Cellular Differentiation Mechanism. Dev. Cell.

[9] Kambe T., Tsuji T., Hashimoto A., Itsumura N. (2015). The Physiological, Biochemical, and Molecular Roles of Zinc Transporters in Zinc Homeostasis and Metabolism. Physiol. Rev..

[10] Jaffe E.K. (2016). The Remarkable Character of Porphobilinogen Synthase. Acc. Chem. Res..

[11] Krishnamurthy V.M., Kaufman G.K., Urbach A.R., Gitlin I., Gudiksen K.L., Weibel D.B., Whitesides G.M. (2008). Carbonic Anhydrase as a Model for Biophysical and Physical-Organic Studies of Proteins and Protein−Ligand Binding. Chem. Rev..

[12] King J.C. (2011). Zinc: An Essential but Elusive Nutrient. Am. J. Clin. Nutr..

[13] Kumssa D.B., Joy E.J.M., Ander E.L., Watts M.J., Young S.D., Walker S., Broadley M.R. (2015). Dietary Calcium and Zinc Deficiency Risks Are Decreasing but Remain Prevalent. Sci. Rep..

[14] Wessells K.R., Brown K.H. (2012). Estimating the Global Prevalence of Zinc Deficiency: Results Based on Zinc Availability in National Food Supplies and the Prevalence of Stunting. PLoS ONE.

[15] King L.E., Fraker P.J. (2002). Zinc Deficiency in Mice Alters Myelopoiesis and Hematopoiesis. J. Nutr..

[16] King L.E., Frentzel J.W., Mann J.J., Fraker P.J. (2005). Chronic Zinc Deficiency in Mice Disrupted T Cell Lymphopoiesis and Erythropoiesis While B Cell Lymphopoiesis and Myelopoiesis Were Maintained. J. Am. Coll. Nutr..

[17] Gibson R.S., Abebe Y., Stabler S., Allen R.H., Westcott J.E., Stoecker B.J., Krebs N.F., Hambidge K.M. (2008). Zinc, Gravida, Infection, and Iron, but Not Vitamin B-12 or Folate Status, Predict Hemoglobin during Pregnancy in Southern Ethiopia. J. Nutr..

[18] Houghton L.A., Parnell W.R., Thomson C.D., Green T.J., Gibson R.S. (2016). Serum Zinc Is a Major Predictor of Anemia and Mediates the Effect of Selenium on Hemoglobin in School-Aged Children in a Nationally Representative Survey in New Zealand. J. Nutr..

[19] Atasoy H.I., Bugdayci G. (2018). Zinc Deficiency and Its Predictive Capacity for Anemia: Unique Model in School Children. Pediatr. Int..

[20] Prasad A.S., Halsted J.A., Nadimi M. (1961). Syndrome of Iron Deficiency Anemia, Hepatosplenomegaly, Hypogonadism, Dwarfism and Geophagia. Am. J. Med..

[21] Prasad A.S., Miale A., Farid Z., Sandstead H.H., Schulert A.R. (1963). Zinc Metabolism in Patients with the Syndrome of Iron Deficiency Anemia, Hepatosplenomegaly, Dwarfism, and Hypognadism. J. Lab. Clin. Med..

[22] Grillo A.S., SantaMaria A.M., Kafina M.D., Cioffi A.G., Huston N.C., Han M., Seo Y.A., Yien Y.Y., Nardone C., Menon A.V. (2017). Restored Iron Transport by a Small Molecule Promotes Absorption and Hemoglobinization in Animals. Science.

[23] Ebert P.S., Hess R.A., Frykholm B.C., Tschudy D.P. (1979). Succinylacetone, a Potent Inhibitor of Heme Biosynthesis: Effect on Cell Growth, Heme Content and δ-Aminolevulinic Acid Dehydratase Activity of Malignant Murine Erythroleukemia Cells. Biochem. Biophys. Res. Commun..

[24] Guggisberg C.A., Kim J., Lee J., Chen X., Ryu M.-S. (2022). NCOA4 Regulates Iron Recycling and Responds to Hepcidin Activity and Lipopolysaccharide in Macrophages. Antioxidants.

[25] Parsons P.J. (2001). Measurement of Erythrocyte Protoporphyrin Concentration by Double Extraction and Spectrofluorometry. Curr. Protoc. Toxicol..

[26] Hanna T.L., Dietzler D.N., Smith C.H., Gupta S., Zarkowsky H.S. (1976). Erythrocyte Porphyrin Analysis in the Detection of Lead Poisoning in Children: Evaluation of Four Micromethods. Clin. Chem..

[27] Garden J.S., Mitchell D.G., Jackson K.W., Aldous K.M. (1977). Improved Ethanol Extraction Procedure for Determining Zinc Protoporphyrin in Whole Blood. Clin. Chem..

[28] Ye J., Coulouris G., Zaretskaya I., Cutcutache I., Rozen S., Madden T.L. (2012). Primer-BLAST: A Tool to Design Target-Specific Primers for Polymerase Chain Reaction. BMC Bioinform..

[29] Hardyman J.E.J., Tyson J., Jackson K.A., Aldridge C., Cockell S.J., Wakeling L.A., Valentine R.A., Ford D. (2016). Zinc Sensing by Metal-Responsive Transcription Factor 1 (MTF1) Controls Metallothionein and ZnT1 Expression to Buffer the Sensitivity of the Transcriptome Response to Zinc. Metallomics.

[30] Langmade S.J., Ravindra R., Daniels P.J., Andrews G.K. (2000). The Transcription Factor MTF-1 Mediates Metal Regulation of the Mouse ZnT1 Gene. J. Biol. Chem..

[31] Philpott C.C., Ryu M.-S. (2014). Special Delivery: Distributing Iron in the Cytosol of Mammalian Cells. Front. Pharmacol..

[32] Iwai K., Klausner R.D., Rouault T.A. (1995). Requirements for Iron-Regulated Degradation of the RNA Binding Protein, Iron Regulatory Protein 2. EMBO J..

[33] Welch J.J. (2004). Global Regulation of Erythroid Gene Expression by Transcription Factor GATA-1. Blood.

[34] Bloomer J.R., Reuter R.J., Morton K.O., Wehner J.M. (1983). Enzymatic Formation of Zinc-Protoporphyrin by Rat Liver and Its Potential Effect on Hepatic Heme Metabolism. Gastroenterology.

[35] Rettmer R.L., Carlson T.H., Origenes M.L., Md J., Jack R.M., Labbé R.F. (1999). Zinc Protoporphyrin/Heme Ratio for Diagnosis of Preanemic Iron Deficiency. Pediatrics.

[36] Hastka J., Lasserre J.J., Schwarzbeck A., Hehlmann R. (1994). Central Role of Zinc Protoporphyrin in Staging Iron Deficiency. Clin. Chem..

[37] Siyame E.W.P., Hurst R., Wawer A.A., Young S.D., Broadley M.R., Chilimba A.D.C., Ander L.E., Watts M.J., Chilima B., Gondwe J. (2013). A High Prevalence of Zinc- but Not Iron-Deficiency among Women in Rural Malawi: A Cross-Sectional Study. Int. J. Vitam. Nutr. Res..

[38] Killilea D.W., Rohner F., Ghosh S., Otoo G.E., Smith L., Siekmann J.H., King J.C. (2017). Identification of a Hemolysis Threshold That Increases Plasma and Serum Zinc Concentration. J. Nutr..

[39] Chen Y.-H., Jeng S.-S., Hsu Y.-C., Liao Y.-M., Wang Y.-X., Cao X., Huang L.-J. (2020). In Anemia Zinc Is Recruited from Bone and Plasma to Produce New Red Blood Cells. J. Inorg. Biochem..

[40] Ryu M.-S., Lichten L.A., Liuzzi J.P., Cousins R.J. (2008). Zinc Transporters ZnT1 (Slc30a1), Zip8 (Slc39a8), and Zip10 (Slc39a10) in Mouse Red Blood Cells Are Differentially Regulated during Erythroid Development and by Dietary Zinc Deficiency. J. Nutr..

[41] Faraji B., Swendseid M.E. (1983). Growth Rate, Tissue Zinc Levels and Activities of Selected Enzymes in Rats Fed a Zinc-Deficient Diet by Gastric Tube. J. Nutr..

[42] Abdulla M., Haeger-Aronsen B., Mathur A., Wallenius K. (1978). Effect of Age and Diet on Delta-Aminolevulinic AcidDehydratase in Red Blood Cells. Enzyme.

[43] King J.C., Brown K.H., Gibson R.S., Krebs N.F., Lowe N.M., Siekmann J.H., Raiten D.J. (2015). Biomarkers of Nutrition for Development (BOND)-Zinc Review. J. Nutr..

[44] Thurnham D., Northrop-Clewes C. (2013). Biomarkers for the Differentiation of Anemia and Their Clinical Usefulness. J Blood Med.

