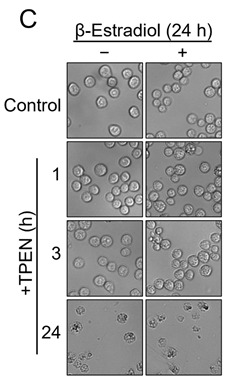# Correction: Kim et al. Cellular Zinc Deficiency Impairs Heme Biosynthesis in Developing Erythroid Progenitors. *Nutrients* 2023, *15*, 281

**DOI:** 10.3390/nu17152402

**Published:** 2025-07-23

**Authors:** Juyoung Kim, Jaekwon Lee, Moon-Suhn Ryu

**Affiliations:** 1 Department of Food Science and Nutrition, University of Minnesota, St. Paul, MN 55108, USA; 2 Department of Biochemistry, University of Nebraska-Lincoln, Lincoln, NE 68588, USA; 3 Department of Food and Nutrition, Yonsei University, Seoul 03722, Republic of Korea

In the original publication [1], there was a mistake in Figure 1C as published. Specifically, the image of cells treated with TPEN for 1 h under the presence of a 24 h treatment of β-estradiol was mistakenly replaced by a duplicated image of cells treated with TPEN for 1 h without any β-estradiol. The corrected Figure 1C appears below. The authors state that the scientific conclusions are unaffected. This correction was approved by the Academic Editor. The original publication has also been updated.